# Biological Effects of Food Coloring in In Vivo and In Vitro Model Systems

**DOI:** 10.3390/foods8050176

**Published:** 2019-05-24

**Authors:** Rocío Merinas-Amo, María Martínez-Jurado, Silvia Jurado-Güeto, Ángeles Alonso-Moraga, Tania Merinas-Amo

**Affiliations:** Department of Genetics, University of Córdoba, 14071 Córdoba, Spain; rocio.merinas@gmail.com (R.M.-A.); martinezjurado.maria@gmail.com (M.M.-J.); silviajuradogueto@gmail.com (S.J.-G.); ge1almoa@uco.es (Á.A.-M.)

**Keywords:** additives, food coloring, *Drosophila melanogaster*, leukemia cells, toxicity, antitoxicity, longevity, cytotoxicity, DNA damage, methylation status

## Abstract

(1) Background: The suitability of certain food colorings is nowadays in discussion because of the effects of these compounds on human health. For this reason, in the present work, the biological effects of six worldwide used food colorings (Riboflavin, Tartrazine, Carminic Acid, Erythrosine, Indigotine, and Brilliant Blue FCF) were analyzed using two model systems. (2) Methods: In vivo toxicity, antitoxicity, and longevity assays using the model organism *Drosophila melanogaster* and in vitro cytotoxicity, DNA fragmentation, and methylation status assays using HL-60 tumor human cell line were carried out. (3) Results: Our in vivo results showed safe effects in *Drosophila* for all the food coloring treatments, non-significant protective potential against an oxidative toxin, and different effects on the lifespan of flies. The in vitro results in HL-60 cells, showed that the tested food colorings increased tumor cell growth but did not induce any DNA damage or modifications in the DNA methylation status at their acceptable daily intake (ADI) concentrations. (4) Conclusions: From the in vivo and in vitro studies, these results would support the idea that a high chronic intake of food colorings throughout the entire life is not advisable.

## 1. Introduction

A food coloring is a dye, pigment, or substance that, when added to food, drugs, or cosmetics, is able to provide color. The Food and Drugs Administration (FDA) is responsible for regulating dyes to assure their safety. Dyes are classified on the basis of their necessity of certification. According to the FDA, dyes are used to confer color to food that has lost it and to improve the color or provide it to uncolored food to make it attractive [1].

A food additive is defined as “any substance not normally consumed as food by itself and not normally used as a typical ingredient of food, whether or not it has nutritive value, the intentional addition of which to food for a technological (including organoleptic) purpose in the manufacture, processing, preparation, treatment, packing, packaging, transport, or holding of such food results, or may be reasonably expected to result (directly or indirectly), in it or its by-products becoming a component or otherwise affecting the characteristics of such food” [2].

Additives are found in many types of food that we often consume not knowing that they are present, so it is very important to study the biological consequences of using food coloring. Moreover, because of the well-known relationship between diet and health and the increasing awareness of people about their quality of life, a great deal of studies have been performed to determine which dyes may be harmful for health, promoting, for instance, childhood hyperactivity, urticaria, asthma [3], and rhinitis [4]. Information about the most consumed food coloring is reported below:

- Riboflavin (E-101) is part of the vitamin B group. It is a yellow-orange solid substance with poor solubility in water. This food coloring is present in a wide range of foods, with liver, milk, meat, and fish being the most important sources [5]. Riboflavin can be obtained by controlled fermentation using a genetically modified strain of *Bacillus subtilis* or the fungus *Ashbya gossypii* [6]. Riboflavin was evaluated by the Joint FAO/WHO Expert Committee on Food Additives (JECFA) in 1969, which established an acceptable daily intake (ADI) of 0.5 mg/kg·body weight (bw)/day on the basis of limited data [5]. No adverse toxic, genotoxic, cytotoxic, or allergic effects have been related to Riboflavin in different organisms [7,8].
- Tartrazine (E-102) is a synthetic lemon-yellow azo dye primarily used as a food coloring. Its presence is allowed in various foodstuffs and beverages [9]. Both the JECFA and the EU Scientific Committee for Food (SCF) established an ADI of 7.5 mg/kg·bw/day in 1996 [10]. Controversial studies about the effects of Tartrazine on health have been reported. The most adverse effects have been related to DNA damage [11], hyperactivity [12], changes in the central nervous system [13], and allergic reactions [14,15,16,17,18].
- Carminic Acid (E-120) is a natural red colorant which comes from *Datylopius coccus*, an insect which lives on *Opuntia coccinellifer*. In order to obtain this dye, it is necessary to dry and spray the body of pregnant females of these insects [19]. The JECFA and SCF committees established an ADI of 5 mg/kg·bw/day for Carminic Acid [20]. This dye is called by the FDA “cochineal extract” or “carmine” and is classified as exempt from certification. According to the FDA, it is used in food, drugs, and cosmetics [1]. Despite the absence of genotoxic or cytotoxic effects described for Carminic Acid, it has been related to anaphylactic reactions, asthma, urticaria, and angioedema [19,21,22,23]. Furthermore, impairment in renal function has been demonstrated in male albino rats [24].
- Erythrosine (E-127) is a cherry-pink synthetic food colorant with a polyiodinated xanthene structure [25]. It is widely used to color children’s sweets [26], as well as to determine the presence of dental plate in Odontology [27]. The ADI of Erythrosine was established by the JECFA and SCF in 0.1 mg/kg·bw/day [28]. Regarding the FDA, it allows the use of Erythrosine both for food and drugs [1]. Some studies suggested a relationship between Erythrosine consumption and altered cognition and behavior in children, which could be due to the inhibition of dopamine receptors [29]. Moreover, different studies suggested the induction of chromosome aberrations and an increase in the incidence of thyroid tumors by Erythrosine consumption [11,30,31].
- Indigotine (E-132) is one of the earliest known natural dyes. Originally, it was obtained from the leaves of the plants *Indigofera tinctoria*, *Indigofera suifruticosa*, and *Isatis tinctoria*, where it occurs as indican, a glycoside of indoxyl [32]. In 1975, the JECFA and SCF established an ADI of 5 mg/kg·bw/day for this blue additive [33]. Only a subacute toxicity study performed with adult male Swiss albino mice showed severe adverse effects of Indigotine on the testis [34].
- Brilliant Blue FCF (E-133) is a triarylmethane synthetic food coloring authorized as a food additive. In 2017, the JECFA revised the ADI to 6 mg/kg·bw/day for this blue additive [35]. Brilliant Blue FCF has recently been evaluated and approved as a cosmetic colorant by the Scientific Committee on Cosmetic Products (SCCP) [35]. Current databases show no adverse effects of Brilliant Blue FCF in any organism assayed for any biological test carried out [11,36,37,38,39].

Considering the available information about the toxicological effects of food coloring on health, our main goal was to evaluate the biological and nutritional effects that the mentioned additives have on time-related degenerative processes, as well as to add new scientific data. For that purpose, an integrative study of the biological activity at the individual, cellular, and molecular levels based on in vivo and in vitro assays was carried out using two model systems. The *Drosophila* animal model is known to have more than 75% of human disease homologous genes [40] related to different human degenerative illnesses, such as Parkinson’s and Alzheimer’s diseases, and allergic diseases, among others. For this reason, it is a reliable system to test toxicity, antitoxicity, longevity, and many other processes [41]. Moreover, using an in vitro model of human leukemia cells (HL-60), we studied the effect of this compound on cell growth inhibition, DNA damage (internucleosomal fragmentation as double-strand breaks leading to DNA laddering associated with the activation of the apoptotic pathway in cells), and the modulation of the methylation status. The purpose of the present study was to extend knowledge and provide new scientific data in this area for future clinical studies.

## 2. Materials and Methods

### 2.1. Samples

Six different types of food coloring were selected for this study according to their high consume and abundance in the diet. A range of six concentrations were tested for each food coloring in order to better understand their biological activity at different endpoints in in vivo and in vitro assays.

The concentrations of the food colorings were established taking into account the average daily food intake of *Drosophila melanogaster* (1 mg/day) and the average body weight of *D. melanogaster* individuals (1 mg) [42]. The concentration range for all tested substances was calculated in order to make it comparable with their ADI in humans, as it summarized in Table 1. 

### 2.2. In Vivo Assays

The value of using *Drosophila* to investigate fundamental biological processes is increasingly evident. This organism is revealing itself as an appropriate system as it is a complex multicellular organism in which many aspects of gene expression are parallel to those of humans. *Drosophila* substitute mammals in experiments with the distinct goal of uncovering insights directly relevant to human beings, because it is a model for many human diseases, including cancer and ageing [43,44,45].

In the present study, two *Drosophila* strains were used, each with a hair marker in the third chromosome: (i) *mwh*/*mwh*, carrying the recessive mutation *mwh* (multiple wing hairs) that in homozygosis produces multiple tricomas per cell instead of one per cell [46], and (ii) *flr^3^/In (3LR) TM3, rip^p^sep bx^34e^ e^s^ Bd^S^*, where the *flr^3^* (flare) marker is a homozygous recessive lethal mutation that produces deformed tricomas but is viable in homozygous somatic cells once larvae start development. All in vivo treatments were carried using the offspring of the reciprocal crosses of the two strains, to finally use the emerging trans-heterozygous individuals (*mwh·flr^+^/mwh^+^·flr^3^*) for the different toxicity, antitoxicity, and longevity assays [47]. 

#### 2.2.1. Toxicity and Antitoxicity Assays

The survival percentages of treated *Drosophila* were determined in toxicity assays ((number of individuals born in each treatment group/number of individuals born in the negative control group) × 100). The antitoxicity tests consisted of combining treatments with food colorings at the same concentrations as in the toxicity assays with H_2_O_2_ at 0.12 M (Sigma; H1009) [48]. The negative controls were prepared with *Drosophila* Instant Medium (Formula 4-24, Carolina Biological Supply, Burlington, NC) and distilled water, and the positive controls with medium and H_2_O_2_. 

Three independent experiments were carried out for each assay. Chi-square test in Microsoft Office Excel 2007 was used to determine if the tested compounds significantly affected fly survival, with respect to the control. In the toxicity assay, statistical chi-square values (*p* < 0.05) for the different concentrations tested were obtained by comparing the effects of different concentrations with those of the negative control, whereas statistical chi-square values of antitoxicity assays were obtained by comparing the effects of the different concentrations with those of the positive control [49].

A wide range of researches are found on the effects of hydrogen peroxide: it can interact directly with DNA or modulate transcription and suppress genomic repair pathways; induce microsatellite instability in germ cells of *D. melanogaster* [50]; produce genetic damage due to the generated electrophilic compounds [51]. Also, it is well established that hydrogen peroxide is an endogenous mutagen responsible for some of the highest cancer risks associated with persistent inflammation [52,53]. Oxy-radicals derived from hydrogen peroxide can act on the genome either directly, causing chromosome damage that induces oncogenic mutations [54,55], or indirectly, by modulating gene transcription [56,57] or by suppressing genome repair pathways [58,59]. Moreover, a study of genotoxicity induced by hydrogen peroxide using the in vivo *Drosophila* assay [60] indicated that the oxidative agent is able to induce somatic mutations and mitotic recombination (concentrations ranged from 0.12 M to 0.48 M). The relative contribution of the recombinational events to the total clone induction was estimated by comparing the frequency of *mwh* spots on the marker wings with the frequency of *mwh* spots in the balancer wings, concluding that an average of 60% of clones showed a genetic recombinational origin.

#### 2.2.2. Lifespan Assays

All experiments were carried out at 25 °C according to the procedure described in Tasset-Cuevas, et al. [61]. Sets of 25 individuals of the same gender were selected and placed into sterile vials containing 0.21 g of *Drosophila* Instant Medium and 1 mL of different concentrations of solutions of the food coloring to be tested. Two replicates were followed during the complete life extension for each control and concentration established. Alive animals were counted, and the respective nourishment renewed twice a week.

In order to know the quality of life of the treated *Drosophila* in the longevity trials, the upper 25% of lifespan survival curves was studied. This part of the lifespan is considered as the healthspan of a curve, characterized by low and more or less constant age-specific mortality rate values [62]. 

The statistical treatment of the survival data for each control and concentration was carried out with the SPSS Statistics 17.0 software (SPSS, Inc., Chicago, IL, USA), applying the Kaplan–Meier method to obtain the survival curves. The significance of the curves was determined using the Log-Rank method (Mantel-Cox).

### 2.3. In Vitro Assays

The in vitro model of human leukemia cells (HL-60) was used to study the effect of food coloring on growth inhibition of the tumor cells, DNA damage (internucleosomal fragmentation as double-strand breaks leading to DNA laddering associated with the activation of the apoptotic pathway), and modulation of DNA methylation status.

The promyelocytic human leukemia HL-60 cell line was grown in RPMI-1640 medium (Sigma, R5886) supplemented with heat-inactivated fetal bovine serum (Linus, S01805), L-glutamine at 200 mM (Sigma, G7513), and an antibiotic–antimycotic solution (Sigma, A5955). The cells were incubated at 37 °C in a humidified atmosphere with 5% CO_2_ [63]. The cultures were plated at 2.5 × 10^4^ cells/mL density in 10 mL culture bottles and passed every two days.

#### 2.3.1. Cytotoxicity Assays

HL-60 cells were placed in 96-well culture plates (2 × 10^4^ cells/mL), cultured for 72 h, and supplemented with the food colorings at different concentrations. This allowed the assessment of a wide range of concentrations in the in vitro cytotoxicity assays, with the aim to predict acute in vivo lethality. Although a continuous evaluation of the cytotoxic effects was studied, only the results at 72 h allowed us to acquire more knowledge about the in vitro lethality of the tested food colorings at the different concentrations assayed, because the IC_50_ was reached for most of them at that time-point.

Cell viability was determined by the trypan blue dye exclusion test. Trypan blue (Sigma-Aldrich, St. Louis, MO, USA, T8154) was added to the cell cultures at a 1:1 volume ratio, and 20 μL of cell suspension was loaded into a Neubauer chamber. The cells were counted with an inverted microscope at 100× magnification (AE30/31, Motic). Curves were plotted as the average survival percentage of three independent experiments with respect to the control growing for 72 h. 

#### 2.3.2. Determination of DNA fragmentation

HL-60 cells (1 x 10^6^/mL) were treated with different concentrations of food coloring for 5 h. 

The treated cells were collected and centrifuged at 3000 rpm for 5 min, and DNA was extracted according to the procedure described in Merinas-Amo, et al. [64]. Briefly, the cell pellet was resuspended in lysis buffer and incubated in an SDS 10% and proteinase K solution. DNA precipitation with NaCl and isopropanol was followed by washing with 70% ethanol DNA and incubation with RNAse overnight. For the negative control, RPMI was used as the cell medium; as a routine positive control, a concentration of 62.5 mg/mL of a lyophilized blond beer (LBB) was used [64].

DNA was quantified with a spectrophotometer (Nanodrop ND-1000), and 1200 ng of DNA was subjected to 2% agarose gel electrophoresis at 85 mA for 25 min, stained with GelRed, and visualized under UV light.

#### 2.3.3. Methylation Status

Genomic DNA was isolated in the same way as described in the DNA fragmentation section. Bisulphite-modified DNA from food coloring treatments, using the EZ DNA Methylation-Gold Kit, was used as a template for fluorescence-based real-time quantitative Methylation-Specific PCR (qMSP). qMSP was carried out according to the protocol described by Merinas-Amo, et al. [65] in 48-well plates in a MiniOpticon Real-Time PCR System (MJ Mini Personal Thermal Cycler, Bio-Rad Laboratories Inc., Hercules, CA, USA) and was analyzed by the Bio-Rad CFX Manager 3.1 Software. Briefly, the final reaction mixture with a total volume of 10 µL consisted of: 2 µL of deionized water, 5 µM of each forward and reverse primer, 2 µL of iTaq™ Universal SYBR^®^ Green Supermix (Bio-Rad, containing antibody-mediated hot-start iTaq DNA polymerase, dNTPs, MgCl_2_, SYBR^®^ Green I dye, enhancers, stabilizers, and a blend of passive reference dyes including ROX and fluorescein), and 25 ng of bisulphite-converted genomic DNA. qMSP conditions were as follows: one step at 95 °C for 3 min, 45 cycles at 95 °C for 10 s, 60 °C for 15 s, 72 °C for 15 s, another step at 95 °C for 30 s, followed by a 65 °C step during 30 s and finally a boost step from 65 °C to 95 °C for 95 s, increasing the temperature of 0.5 °C each 0.05 s.

Repetitive elements were selected in order to analyze a wide range of human genomic DNA. While Alu and LINE sequences are interspersed throughout the genome, satellites are confined to the centromere areas [66,67,68,69]. All sequences were obtained from Isogen Life Science. Alu M1, LINE-1, and Sat-α sequences were used (see Table 2 for detailed information) [70].

The relative yield results were normalized with respect to the housekeeping sequence Alu C4 using the Nikolaidis, et al. [71] and Liloglou, et al. [72] comparative C_T_ method:

- C_T_ of the target gene was normalized with respect to the referent gene (ΔC_T_).
- ΔC_T_ of each experimental sample or reference (ΔC_T,r_) were compared with ΔC_T_ of the calibrator sample (ΔC_T,cb_), ΔΔC_T._
- The relative value of each sample was defined using the formula:
  2−(^Δ^C_T,r_−^Δ^C_T,cb_) = 2−^ΔΔ^C_T_

Each sample was analyzed in triplicate. One-way ANOVA and post hoc Tukey’s tests were used to evaluate the differences among the tested compounds, repetitive elements, and concentrations.

## 3. Results

### 3.1. In Vivo

#### 3.1.1. Toxicity and Antitoxicity

**Figure 1:**
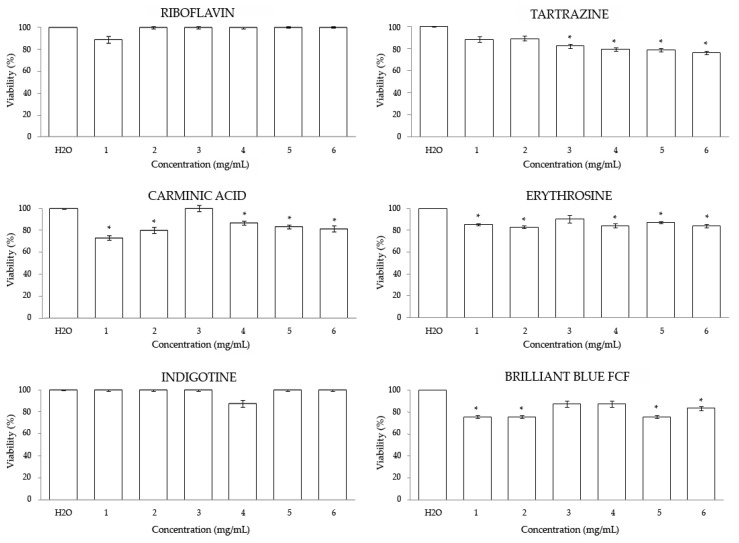
Toxicity levels of food coloring in *Drosophila melanogaster*. Data are expressed as percentage of surviving adults with respect to 300 untreated 72 h-old larvae from three independent experiments treated with different concentrations of food colorings. Values represent the mean ± SE from three independent experiments. * Indicates significant differences with respect to the control. 1– 6 numbers indicate the different dilutions tested (see Table 1).

Figure 1 shows the relative percentage of emerging adults after toxicity treatments with different concentrations of food colorings. Our results showed that Riboflavin and Indigotine were non-toxic at any assayed concentration. Tartrazine showed a significant dose-independent survival percentage at the assayed concentrations, being toxic at the fourth highest concentrations with respect to the control. Moreover, a significant survival rate compared with the control was shown for individuals treated with the red additives, except for the concentration numbered as 3, with a decreasing rate of *Drosophila* survival lower than 80%. Brilliant Blue FCF also showed a significant diminution of the survival of *Drosophila* at the two highest and the two lowest concentrations tested with respect to the control.

On the whole, any food coloring at any assayed concentration reached the lethal dose 50 (LD_50_), which is considered toxic. This fact confirms in the *Drosophila* in vivo eukaryotic model that the ADI (concentration numbered as 3) established by the JECFA for each food coloring is a safe dose [5,10,20,28,33,35].

**Figure 2:**
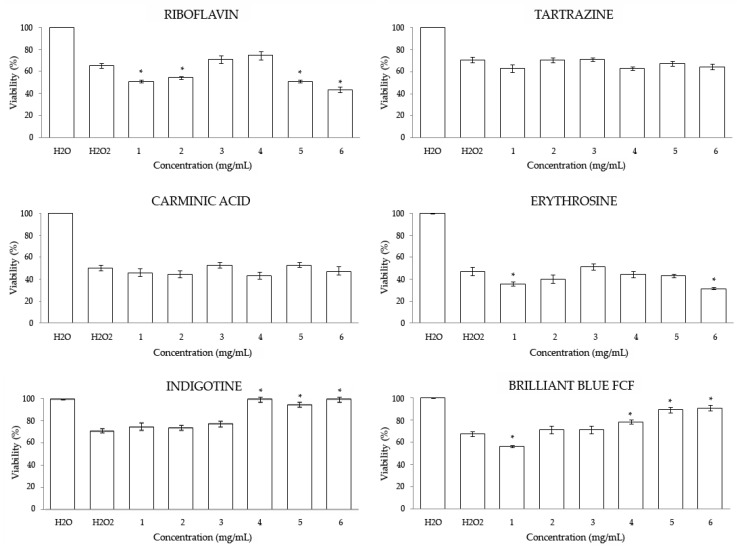
Antitoxicity levels of food coloring in *D. melanogaster*. Data are expressed as percentage of surviving adults with respect to 300 untreated 72 h-old larvae from three independent experiments treated with different concentrations of food colorings combined with 0.12 M H_2_O_2_. Values represent the mean ± SE from three independent experiments. * Indicates significant differences with respect to the positive control. 1–6 numbers indicate the different dilutions tested (see Table 1).

The antitoxicity results showed in Figure 2 revealed the ability of the blue additives to protect individuals against stress, although only at three-highest concentrations assayed. Furthermore, Tartrazine and Carminic Acid showed no significant effects in the combined treatments at any concentrations tested with respect to the positive control. On the other hand, extremes concentrations of Riboflavin and Erythrosine and the lowest concentration of Brilliant Blue FCF showed a toxic synergic effect when combined with the oxidative toxicant hydrogen peroxide in *Drosophila*.

The absence of a relationship between the toxicity and the antitoxicity results in Tartrazine, Carminic Acid, Erythrosine, and Brilliant Blue FCF could be due to the fact that each substance might exhibit antioxidant or prooxidant activities in a competitive manner against the effect of hydrogen peroxide when combined with it [73].

#### 3.1.2. Lifespan

**Figure 3:**
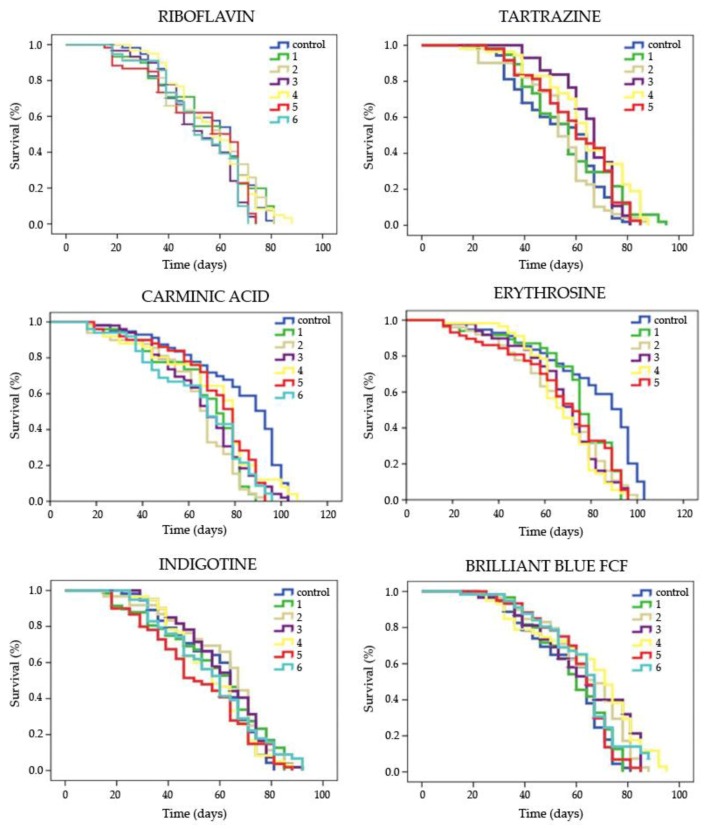
Complete survival curves of *D. melanogaster* fed with different concentrations of food colorings. The numbers 1–6 indicate the different dilutions tested (see Table 1). Curves were obtained by the Kaplan–Meier method, and significance was determined by the Log-Rank method (Mantel-cox).

The entire lifespan curves obtained by the Kaplan–Meier method for each substance and concentration are shown in Figure 3. Tartrazine and Brilliant Blue FCF induced a lifespan extension in *D. melanogaster* at the three highest concentrations tested and at the concentrations numbered 2 to 4, corresponding to 5–10 and 5–8 days, respectively, with respect to their control (Table 3). On the other hand, all concentrations of Carminic Acid and Erythrosine, except the lowest one, showed a significant decrease of longevity corresponding to 9–14 and 12–13 days, respectively, compared with their control, except for the lowest concentration (Table 3). With respect to Riboflavin and Indigotine, no significant effect on *Drosophila* longevity was observed at any assayed concentration.

The healthspan results (upper 25% portion of the lifespan curves) are shown in Table 3. Tartrazine induced a significant increase of healthspan in *D. melanogaster* when compared with the negative control, with the exception of the concentration numbered as 2, whose effect was similar to that of the control. The value of mean survival time of this additive ranged between 4 and 12 days. On the other hand, the highest concentration of Erythrosine assayed and the concentrations numbered 1 and 5 of Indigotine showed a significant reduction in the quality of life of *D. melanogaster* after 16 and 6 days, respectively. The remaining food colorings showed no significant differences in the mean value of healthspan of the treated flies with respect to their concurrent controls.

A non-visible dose–effect relationship has been shown by the different food colorings studied, suggesting a threshold level, rather than a degree of variation, in the significant cohorts. The data provided to the research community by the present study could be related to the controversial results observed in the database about food coloring [5,10,20,28,33,35].

### 3.2. In Vitro

#### 3.2.1. Cytotoxicity

In general, the red and yellow additives showed a dose-dependent response, with an increase of the cytotoxicity level according to the increased concentration of the food coloring. All food colorings reached an inhibitory concentration 50 (IC_50_) between the concentration numbered as 5 and 6 in HL-60 cells, except for Riboflavin that was the only dye able to induce total death of the tumor cells at the concentration numbered as 5. 

**Figure 4:**
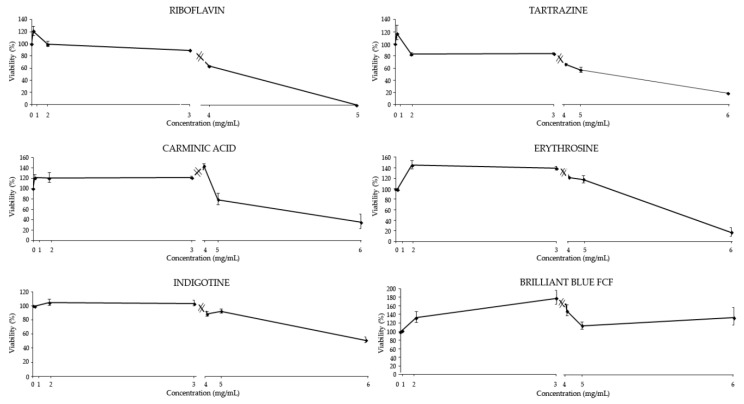
Effect of food coloring on HL-60 cells viability. Viability of the promyelocytic human leukemia cells (HL-60) treated with different concentrations of food colorings for 72 h. Each point represents the growing percentage with respect to its control. Values represent the mean ± SE from three independent experiment. Numbers 1–6 indicate the different dilutions tested (see Table 1).

In relation to the blue additives, Indigotine showed a slight (51%) growth inhibition at the highest concentration tested. No inhibition was observed for Brilliant Blue FCF at any concentration tested, with respect to the control but, contrarily, a tendency to promote cell growth was observed. The concentration numbered as 4 and 5 were the closest to the established ADI for Brilliant Blue FCF; we found that, although their viability-promoting effect was higher than the corresponding effect of the control, it was nonetheless lower than the effect of the other concentrations tested for this food coloring suggesting, their low chemopreventive potential in tumor cells (Figure 4).

#### 3.2.2. DNA Fragmentation

**Figure 5:**
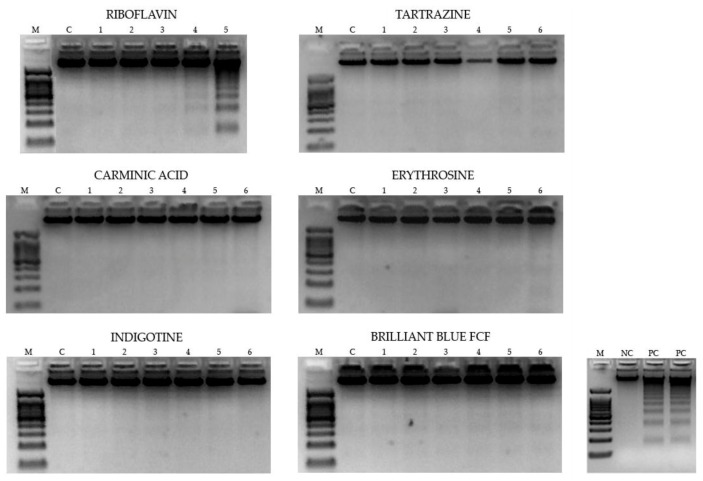
Internucleosomal DNA fragmentation in HL-60 cells. DNA-induced damage in promyelocytic HL-60 cells treated for 5 h with different concentrations of food colorings. M indicates the DNA size marker; NC indicates negative control treatment (RPMI); PC indicates positive control treatment (LBB). Numbers 1–6 indicate the different dilutions tested (see Table 1).

Figure 5 shows electrophoresis experiments of the genomic DNA of HL-60 cells treated with different concentrations of food colorings. The results showed that the proapoptotic hallmark DNA internucleosomal fragmentation was only observed at the highest concentration of Riboflavin assayed. The rest of the food colorings assayed did not induce internucleosomal fragmentation. 

#### 3.2.3. Methylation Status

**Figure 6:**
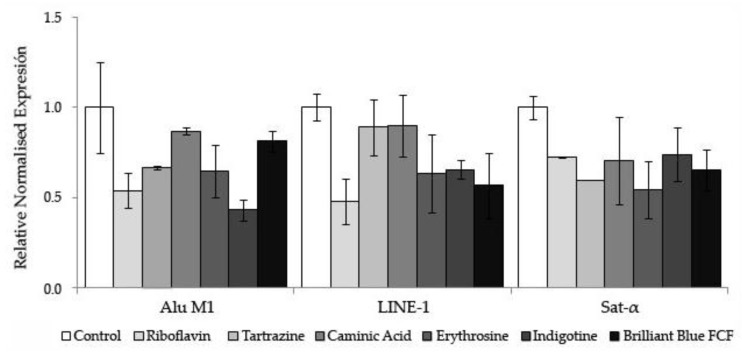
Methylation status of food colorings in HL-60 cells. Relative normalized expression data of each repetitive element (Alu M1, LINE-1, and Sat-α). Values represent the mean ± SE from three independent experiments. Untreated cells grown in RPMI were used as a control.

The relative normalized expression of three repetitive sequences (Alu M1, LINE-1, and Sat-α) studied in HL-60 cells treated with different concentrations of food colorings and RPMI as a control is shown in Figure 6. The food colorings did not modulate the methylation status at the assayed concentrations. After one-way ANOVA and post-hoc Tukey’s test, the statistical results showed a methylation level in the treated samples similar to that of the normalized control.

Despite of the non-significant results in the methylation status for any food coloring tested, Riboflavin exhibited a tendency to hypomethylate the genomic randomly distributed sequences of HL-60 cells (Alu M1 and LINE-1). Taking into account that methylation of repetitive sequences is considered a genomic protective mechanism [70,74], this yellow additive could have inhibitory effects on tumor cells and could be an interesting chemopreventive compound.

## 4. Discussion

### 4.1. In Vivo

According to the toxicity assay, none of the food colorings at any of the assayed concentrations reached the lethal dose 50 (LD_50_) in *D. melanogaster*, which is considered as the toxic level for any substance. Our results are in agreement with the wide variety of researches showing the absence of toxic effects for Riboflavin [75], Tartrazine [11], Carminic Acid [24], Indigotine [76,77,78], and Brilliant Blue FCF [36,79,80] in mice, rats, rabbits, and dogs models. On the other hand, statistically significant severe adverse effects on the testis were described in a subacute toxicity study (45 day) performed on adult male Swiss albino mice treated with oral doses of Indigo of 0, 17, and 39 mg/kg·bw/day [34]. No data were found about Erythrosine toxicity.

Regarding the protective effects of food colorings, no previous data about antioxidative effects were found. Taking into account the concentration corresponding to the equivalent ADI for humans (concentration numbered as 3), no significant results were obtained for any food coloring tested. This fact is in agreement with the results of Scotter and Castle [81], who suggested that, in general, the majority of color additives are unstable in combination with oxidizing and reducing agents in food. Moreover, since color depends on the existence of a conjugated unsaturated system within a dye molecule, any substance which modifies this system (e.g., oxidizing or reducing agents, sugars, acids, and salts) will affect the color [81].

To our knowledge, no previous studies assessing the effects on lifespan and healthspan have been published. Our results indicated that the highest concentrations of Tartrazine and medium quantities of Brilliant Blue FCF induced a significant life extension with respect to the controls, whereas both red food colorings showed significantly negative effects on the longevity of *Drosophila*. Furthermore, quality of life was only improved by Tartrazine and, even, it worsened at some concentrations of Erythrosine and Indigotine. 

On the whole, a non-visible dose–effect relationship for the food colorings could be appreciated in the different assays. This could be explained by the possible differential responses of the organism against each substance and by the biological level at which it was acting.

### 4.2. In Vitro

A dose-dependent cytotoxic effect was observed for the food colorings assayed in HL-60 cells, except for both types of blue dyes which did not reach the inhibitory concentration 50 (IC_50_) or even increased tumor cells’ growth. Our results fit with those that demonstrated that Tartrazine, Carminic Acid, and Erythrosine did not have any potential to induce tumor cells’ growth. The available carcinogenicity studies have demonstrated that Tartrazine does not induce benign or malignant neoplasia [82,83]. Moreover, in a combined chronic toxicity/carcinogenicity study involving in utero exposure of Wistar rats to Carminic Acid, the general pattern of tumor incidence in the treated animals did not significantly differ statistically from those of the controls [84]. Besides, studies about Erythrosine treatments in mice [78], rats [85], and gerbils [86] showed no significant adverse effects of this food coloring. On the other hand, our Indigotine and Brilliant Blue FCF results are not in agreement with those of different researches that indicated no carcinogenetic and tumor potential of this food coloring: exposure of mice to Indigo did not demonstrate carcinogenic or toxic effects [77]; subcutaneous injections of 10 doses of Brilliant Blue FCF, 4 mg each, followed by 50 doses of 6 mg showed no tumor production after 78 weeks in mouse [35]. These controversial results may be due to differences in the organisms used, the cell line studied, or the range of concentrations tested. No data about Riboflavin cytotoxicity were found.

Effects on the DNA damage at the internucleosomal level in HL-60 cells did not appear in our study, with the exception of the highest concentration of Riboflavin. The Indigotine results are supported by in vitro studies using MCF-7 breast cancer cells [87] and the human colonic adenocarcinoma cell line (CaCo2 cells) [88], which demonstrated a lack of statistical significance in DNA damaging. Similar results were obtained in ddY male mice treated with Brilliant Blue FCF, showing not statistically significant increases in DNA damage in glandular stomach, colon, liver, kidney, urinary bladder, lung, brain, and bone marrow [11]. In contrast, our results for Tartrazine and Erythrosine are not in agreement with those of Sasaki, Kawaguchi, Kamaya, Ohshita, Kabasawa, Iwama, Taniguchi, and Tsuda [11] and Tsuda, et al. [89], who demonstrated the effect of Tartrazine on nuclear DNA electrophoretic migration in the mouse and the induction of DNA damage in the stomach at doses of 10 and 2000 mg/kg·bw without a dose–effect relationship, and a dose-related induction of DNA damage by Erythrosine in the glandular stomach, colon, and urinary bladder after oral administration of 100 mg/kg·bw and 2000 mg/kg·bw and in the lung following administration of 2000 mg/kg·bw in mice. To our knowledge, no previous results about the effects of Riboflavin and Carminic Acid on DNA damage were published.

Finally, no significant modification of the DNA methylation status was found compared with the control. This means that modifications of the DNA epigenome are not induced, which is in agreement with studies showing no chromosome aberrations upon Riboflavin [5], Tartrazine [90], and Erythrosine [91] treatments in Chinese hamster ovary cells, mice bone morrow cells, and Syrian Hamster Embryo, respectively.

To sum up, no beneficial effects of food coloring were shown in the different in vitro tests with tumor cells. These controversial data with respect to the current well-known data supporting the safe consumption of additives may be due to the variety of conditions used: cell line, in vitro conditions, range of concentrations, or even the tests conditions.

## 5. Conclusions

Additives are found in many types of food, and we often consume them unknowingly; therefore, it is very important to study the biological consequences of using food coloring. Nowadays, people are becoming more aware of the possible danger of these additives that have no nutritional value.

Two model systems (in vivo and in vitro) were used to carry out the different screening tests. *D. melanogaster* is a well-known insect with a large scientific history in biological sciences that has highly contributed to understanding developmental biology, evolutionary concepts, and, recently, toxicology [92,93,94,95,96]. The unique characteristics that *Drosophila* possesses, such as a rapid and short life cycle (10–12 days at 25 °C), reliability, cost-efficiency, easy maintenance and manipulation, and consistent genetic similarity to humans, make this eukaryote an ideal model organism [40,97]. On the other hand, the human model HL-60 cell line was originated from a female patient with acute myeloid leukemia [98]. The promyelocytic human leukemia cell line HL-60 is used worldwide for many toxicity and cancer scientific purposes [63].

In conclusion, and taking into account the concentration indicated as the equivalent ADI for humans, the in vivo toxicity assays showed safe effects for all food colorings, as shown by the fact that the LD_50_ was not achieved by any of the additives. Nevertheless, no significant differences were shown for any compound in the combined antitoxicity assays with respect to the controls, since they did not protect against oxidative damage by hydrogen peroxide. However, the longevity assays showed a differential behavior of the six food colorings, being Tartrazine and Brilliant Blue FCF the only colorants that significantly improved the longevity of *Drosophila*, whereas the red additives reduced significantly the lifespan of *Drosophila*. On the other hand, the in vitro results demonstrated that, despite the dose-dependent cytotoxic effects shown by the yellow and red additives, none of them reached the IC_50_ at their ADI concentration. Moreover, red and blue food colorings induced an increasing of tumor cell growth. Besides, no DNA damage was observed by the internucleosomal fragmentation apoptotic assay, and no methylation status modification was found for any food coloring. To our knowledge, this is the first time that an integrative study with a wide range of in vivo and in vitro screening tests has been carried out in the model systems *D. melanogaster* and HL-60 tumor cells with food colorings. Several checkpoints to evaluate the biological activity of such important food additives have been established at the molecular (DNA internucleosomal proapoptotic clastogenicity and epigenetic status), unicellular (cytotoxicity), and individual (toxicity, antitoxicity, and longevity) levels. Although more scientific researches are needed to understand the effects that these highly consumed additives could have on our health, these results represent the first step and may encourage additional studies. 

On the whole and despite the safe use suggested by the different assays carried out with food colorings, the overall results would support the idea that a high chronic intake of these additives throughout the entire life is not advisable, and more research on the biological effects that different concentrations of food colorings could have in model systems is warranted. 

## Figures and Tables

**Table 1:** Food coloring information.

Food Coloring		ADI (Mg/Kg)	Tested Concentrations in *Drosophila* (mg/mL) *					
			1	2	3	4	5	6
E-101	Riboflavin	0.5	0.0000025	0.000025	0.00025	0.0025	0.025	0.25
E-102	Tartrazine	7.5	0.0000375	0.000375	0.00375	0.0375	0.375	3.75
E-120	Carminic Acid	5	0.000025	0.00025	0.0025	0.025	0.25	2.5
E-127	Erythrosine	0.1	0.0000005	0.000005	0.00005	0.0005	0.005	0.05
E-132	Indigotine	5	0.000025	0.00025	0.0025	0.025	0.25	2.5
E-133	Brilliant Blue FCF	6	0.0000005	0.000005	0.00005	0.0005	0.005	0.05

* numbers 1 to 6 represent the value, in mg/mL, of the different dilutions assayed in the in vivo and in vitro assays for each food coloring; the concentration corresponding to number 3 is the equivalent quantity of ADI in humans.

**Table 2:** Primers information.

Reaction ID	GenBank Number	Amplicon Start	Amplicon End	Forward Primer Sequence 5’ to 3’ (N)	Reverse Primer Sequence 5’ to 3’ (N)	GC Content (%)	
						Forward Reverse	
Alu C4	Consensus Sequence	1	98	GGTTAGGTATAGTGGTTTATATTTGTAATTTTAGTA (36)	ATTAACTAAACTAATCTTAAACTCCTAACCTCA (33)	25	27.3
Alu M1	Y07755	5059	5164	ATTATGTTAGTTAGGATGGTTTCGATTTT (29)	CAATCGACCGAACGCGA (17)	27.6	58.8
LINE-1	X52235	251	331	GGACGTATTTGGAAAATCGGG (21)	AATCTCGCGATACGCCGTT (19)	47.6	52.6
Sat-α	M38468	139	260	TGATGGAGTATTTTTAAAATATACGTTTTGTAGT (34)	AATTCTAAAAATATTCCTCTTCAATTACGTAAA (33)	23.5	21.2

Source: Weisenberger, Campan, Long, Kim, Woods, Fiala, Ehrlich and Laird [70].

**Table 3:** Mean and significances of lifespan and healthspan curves.

Food coloring	Concentration	Mean Lifespan ^1^ (days)		Mean Healthspan ^1^ (days)	
Riboflavin	Control	55.985		31.399	
	1	55.019	ns	27.607	ns
	2	55.864	ns	29.110	ns
	3	52.534	ns	29.966	ns
	4	57.067	ns	32.714	ns
	5	53.341	ns	25.500	ns
	6	52.660	ns	27.222	ns
Tartrazine	Control	54.375		31.399	
	1	57.664	ns	36.154	*
	2	54.037	ns	32.681	ns
	3	64.618	*	43.571	*
	4	63.860	*	35.760	*
	5	59.989	*	37.252	*
Carminic Acid	Control	62.345		38.509	
	1	44.958	*	35.630	ns
	2	43.215	*	29.000	ns
	3	45.515	*	37.067	ns
	4	46.998	*	39.320	ns
	5	48.211	*	40.000	ns
	6	44.236	*	30.530	ns
Erythrosine	Control	62.345		38.015	
	1	55.487	ns	27.614	ns
	2	49.589	*	34.276	ns
	3	49.847	*	35.051	ns
	4	50.011	*	43.333	ns
	5	50.214	*	22.501	*
Indigotine	Control	58.433		32.988	
	1	57.791	ns	27.019	*
	2	61.547	ns	29.182	ns
	3	61.181	ns	33.857	ns
	4	57.067	ns	32.714	ns
	5	52.024	ns	27.189	*
	6	57.570	ns	31.068	ns
Brilliant Blue FCF	Control	57.526		32.988	
	1	58.686	ns	34.333	ns
	2	63.513	*	31.286	ns
	3	62.664	*	33.000	ns
	4	65.095	*	32.877	ns
	5	61.074	ns	30.800	ns
	6	62.466	ns	31.984	ns

Means were calculated by the Kaplan–Meier method, and significance of the curves was determined by the Log-Rank method (Mantel-Cox). ^1^ ns: non-significant, * significant (*p* < 0.05); numbers 1–6 indicate the different dilutions tested (see Table 1).

## References

[1] Food and Drug Administration (FDA). https://www.fda.gov/1.

[2] Swaroop V., Roy D.D., Vijayakumar T. (2011). Genotoxicity of synthetic food colorants. J. Food Sci. Eng..

[3] Juhlin L., Michaëlsson G., Zetterström O. (1972). Urticaria and asthma induced by food-and-drug additives in patients with aspirin hypersensitivity. J. Allergy Clin. Immunol..

[4] Vedanthan P.K., Menon M.M., Bell T.D., Bergin D. (1977). Aspirin and tartrazine oral challenge: Incidence of adverse response in chronic childhood asthma. J. Allergy Clin. Immunol..

[5] EFSA (2013). Scientific Opinion on the Re-Evaluation of Riboflavin (e 101 (i)) and Riboflavin-5′-Phosphate Sodium (e 101 (ii)) as Food Additives. EFSA J..

[6] Elmadfa I. (2009). European Nutrition and Health Report 2009.

[7] Kale H., Harikumar P., Kulkarni S., Nair P., Netrawali M. (1992). Assessment of the genotoxic potential of riboflavin and lumiflavin: B. Effect of light. Mutat. Res. Genet. Toxicol..

[8] Unna K., Greslin J.G. (1942). Studies on the toxicity and pharmacology of riboflavin. J. Pharmacol. Exp. Ther..

[9] Komisyonu A. (1994). European parliament and council directive 94/36/ec of 30 june 1994 on colours for use in foodstuffs. Off. J. Eur. Union L.

[10] EFSA (2009). Scientific opinion on the re-evaluation tartrazine (e 102). EFSA J..

[11] Sasaki Y.F., Kawaguchi S., Kamaya A., Ohshita M., Kabasawa K., Iwama K., Taniguchi K., Tsuda S. (2002). The comet assay with 8 mouse organs: Results with 39 currently used food additives. Mutat. Res. Genet. Toxicol. Environ. Mutagenesis.

[12] McCann D., Barrett A., Cooper A., Crumpler D., Dalen L., Grimshaw K., Kitchin E., Lok K., Porteous L., Prince E. (2007). Food additives and hyperactive behaviour in 3-year-old and 8/9-year-old children in the community: A randomised, double-blinded, placebo-controlled trial. Lancet.

[13] Novembre E., Dini L., Bernardini R., Resti M., Vierucci A. (1992). Unusual reactions to food additives. Pediatr. Med. Chir. Med. Surg. Pediatrics.

[14] Elhkim M.O., Héraud F., Bemrah N., Gauchard F., Lorino T., Lambré C., Frémy J.M., Poul J.M. (2007). New considerations regarding the risk assessment on tartrazine: An update toxicological assessment, intolerance reactions and maximum theoretical daily intake in france. Regul. Toxicol. Pharmacol..

[15] Bhatia M.S. (2000). Allergy to tartrazine in psychotropic drugs. J. Clin. Psychiatry.

[16] Nettis E., Colanardi M., Ferrannini A., Tursi A. (2003). Suspected tartrazine-induced acute urticaria/angioedema is only rarely reproducible by oral rechallenge. Clin. Exp. Allergy.

[17] Worm M., Vieth W., Ehlers I., Sterry W., Zuberbier T. (2001). Increased leukotriene production by food additives in patients with atopic dermatitis and proven food intolerance. Clin. Exp. Allergy.

[18] Inomata N., Osuna H., Fujita H., Ogawa T., Ikezawa Z. (2006). Multiple chemical sensitivities following intolerance to azo dye in sweets in a 5-year-old girl. Allergol. Int..

[19] Wüthrich B., Kägi M., Stücker W. (1997). Anaphylactic reactions to ingested carmine (e120). Allergy.

[20] EFSA (2015). Scientific opinion on the re-evaluation of cochineal, carminic acid, carmines (e 120) as a food additive. EFSA J..

[21] Sarıkaya R., Selvi M., Erkoç F. (2012). Evaluation of potential genotoxicity of five food dyes using the somatic mutation and recombination test. Chemosphere.

[22] DiCello M.C., Myc A., Baker J.R., Baldwin J.L. (1999). Anaphylaxis after Ingestion of Carmine Colored Foods: Two Case Reports and a Review of the Literature, Allergy and Asthma Proceedings.

[23] Ferrer Ð., Marco F.M., Andreu C., Sempere J.M. (2005). Occupational asthma to carmine in a butcher. Int. Arch. Allergy Immunol..

[24] Helal E.G., Zaahkouk S.A., Mekkawy H.A. (2000). Effect of some food colorants (synthetic and natural products) of young albino rats. I–liver and kidney functions. Egypt. J. Hosp. Med..

[25] Chequer F.M.D., de Paula Venâncio V., Bianchi M.d.L.P., Antunes L.M.G. (2012). Genotoxic and mutagenic effects of erythrosine b, a xanthene food dye, on hepg2 cells. Food Chem. Toxicol..

[26] Tuormaa T.E. (1994). The adverse effects of food additives on health: A review of the literature with a special emphasis on childhood hyperactivity. J. Orthomol. Med..

[27] Mpountoukas P., Pantazaki A., Kostareli E., Christodoulou P., Kareli D., Poliliou S., Mourelatos C., Lambropoulou V., Lialiaris T. (2010). Cytogenetic evaluation and DNA interaction studies of the food colorants amaranth, erythrosine and tartrazine. Food Chem. Toxicol..

[28] EFSA (2011). Scientific opinion on the re-evaluation of erythrosine (e 127) as a food additive. EFSA J..

[29] Ganesan L., Margolles-Clark E., Song Y., Buchwald P. (2011). The food colorant erythrosine is a promiscuous protein–protein interaction inhibitor. Biochem. Pharmacol..

[30] Hagiwara M., Watanabe E., Barrett J.C., Tsutsui T. (2006). Assessment of genotoxicity of 14 chemical agents used in dental practice: Ability to induce chromosome aberrations in syrian hamster embryo cells. Mutat. Res. Genet. Toxicol. Environ. Mutagenesis.

[31] Mekkawy H.A., Massoud A., El-Zawahry A. (2000). Mutagenic effects of the food color erythrosine in rats. Probl. Forensic Sci..

[32] Steingruber E. (2000). Indigo and indigo colorants. Ullmann’s Encyclopedia of Industrial Chemistry.

[33] EFSA (2014). Scientific opinion on the re-evaluation of indigo carmine (e 132) as a food additive. EFSA J..

[34] Dixit A., Goyal R. (2013). Evaluation of reproductive toxicity caused by indigo carmine on male swiss albino mice. Pharmacology.

[35] EFSA (2010). Scientific opinion on the re-evaluation of brilliant blue fcf (e 133) as a food additive. EFSA J..

[36] Aboel-Zahab H., El-Khyat Z., Sidhom G., Awadallah R., Abdel-Al W., Mahdy K. (1997). Physiological effects of some synthetic food colouring additives on rats. Boll. Chim. Farm..

[37] Lau K., McLean W.G., Williams D.P., Howard C.V. (2005). Synergistic interactions between commonly used food additives in a developmental neurotoxicity test. Toxicol. Sci..

[38] Borzelleca J., Depukat K., Hallagan J. (1990). Lifetime toxicity/carcinogenicity studies of fd & c blue no. 1 (brilliant blue fcf) in rats and mice. Food Chem. Toxicol..

[39] Lucarelli M.R., Shirk M.B., Julian M.W., Crouser E.D. (2004). Toxicity of food drug and cosmetic blue no. 1 dye in critically ill patients. Chest.

[40] Bier E. (2005). *Drosophila*, the golden bug, emerges as a tool for human genetics. Nat. Rev. Genet..

[41] Graf U., Würgler F., Katz A., Frei H., Juon H., Hall C., Kale P. (1984). Somatic mutation and recombination test in *Drosophila melanogaster*. Environ. Mutagenesis.

[42] Ja W.W., Carvalho G.B., Mak E.M., de la Rosa N.N., Fang A.Y., Liong J.C., Brummel T., Benzer S. (2007). Prandiology of *Drosophila* and the cafe assay. Proc. Natl. Acad. Sci. USA.

[43] Gonzalez C. (2013). *Drosophila melanogaster*: A model and a tool to investigate malignancy and identify new therapeutics. Nat. Rev. Cancer.

[44] Lints F.A., Soliman M.H. (1988). Drosophila as a Model Organism for Ageing Studies.

[45] Rudrapatna V.A., Cagan R.L., Das T.K. (2012). *Drosophila* cancer models. Dev. Dyn..

[46] Yan J., Huen D., Morely T., Johnson G., Gubb D., Roote J., Adler P.N. (2008). The multiple-wing-hairs gene encodes a novel gbd-fh3 domain-containing protein that functions both prior to and after wing hair initiation. Genetics.

[47] Ren N., Charlton J., Adler P.N. (2007). The flare gene, which encodes the aip1 protein of *Drosophila*, functions to regulate f-actin disassembly in pupal epidermal cells. Genetics.

[48] Anter J., Campos-Sanchez J., Hamss R.E., Rojas-Molina M., Munoz-Serrano A., Analla M., Alonso-Moraga A. (2010). Modulation of genotoxicity by extra-virgin olive oil and some of its distinctive components assessed by use of the *Drosophila* wing-spot test. Mutat. Res..

[49] Merinas-Amo T., Tasset-Cuevas I., Díaz-Carretero A.M., Alonso-Moraga A., Calahorro F. (2017). Role of choline in the modulation of degenerative processes: In vivo and in vitro studies. J. Med. Food.

[50] López A., Xamena N., Marcos R., Velázquez A. (2002). Germ cells microsatellite instability: The effect of different mutagens in a mismatch repair mutant of *Drosophila* (spel1). Mutat. Res. Genet. Toxicol. Environ. Mutagenesis.

[51] Allen R., Tresini M. (2000). Oxidative stress and gene regulation. Free Radic. Biol. Med..

[52] Veal E.A., Day A.M., Morgan B.A. (2007). Hydrogen peroxide sensing and signaling. Mol. Cell.

[53] Fitzpatrick F.A. (2001). Inflammation, carcinogenesis and cancer. Int. Immunopharmacol..

[54] Burcham P.C. (1998). Genotoxic Lipid Peroxidation Products: Their DNA Damaging Properties and Role in Formation of Endogenous DNA Adducts.

[55] Feig D.I., Reid T.M., Loeb L.A. (1994). Reactive oxygen species in tumorigenesis. Cancer Res..

[56] Vivancos A.P., Castillo E.A., Biteau B., Nicot C., Ayte J., Toledano M.B., Hidalgo E. (2005). A cysteine-sulfinic acid in peroxiredoxin regulates h2o2-sensing by the antioxidant pap1 pathway. Proc. Natl. Acad. Sci. USA.

[57] Cerda S., Weitzman S. (1997). Influence of oxygen radical injury on DNA methylation. Mutat. Res. Rev. Mutat. Res..

[58] Ghosh R., Mitchell D.L. (1999). Effect of oxidative DNA damage in promoter elements on transcription factor binding. Nucleic Acids Res..

[59] Hu J.J., Dubin N., Kurland D., Ma B.L., Roush G.C. (1995). The effects of hydrogen peroxide on DNA repair activities. Mutat. Res..

[60] Romero-Jiménez M., Campos-Sánchez J., Analla M., Muñoz-Serrano A., Alonso-Moraga Á. (2005). Genotoxicity and anti-genotoxicity of some traditional medicinal herbs. Mutat. Res. Genet. Toxicol. Environ. Mutagenesis.

[61] Tasset-Cuevas I., Fernandez-Bedmar Z., Lozano-Baena M.D., Campos-Sanchez J., de Haro-Bailon A., Munoz-Serrano A., Alonso-Moraga A. (2013). Protective effect of borage seed oil and gamma linolenic acid on DNA: In vivo and in vitro studies. PLoS ONE.

[62] Soh J.W., Hotic S., Arking R. (2007). Dietary restriction in *Drosophila* is dependent on mitochondrial efficiency and constrained by pre-existing extended longevity. Mech. Ageing Dev..

[63] Gallagher R., Collins S., Trujillo J., McCredie K., Ahearn M., Tsai S., Metzgar R., Aulakh G., Ting R., Ruscetti F. (1979). Characterization of the continuous, differentiating myeloid cell line (hl-60) from a patient with acute promyelocytic leukemia. Blood.

[64] Merinas-Amo T., Tasset-Cuevas I., Díaz-Carretero A.M., Alonso-Moraga Á., Calahorro F. (2016). In vivo and in vitro studies of the role of lyophilised blond lager beer and some bioactive components in the modulation of degenerative processes. J. Funct. Foods.

[65] Merinas-Amo T., Merinas-Amo R., Alonso-Moraga A. (2017). A clinical pilot assay of beer consumption: Modulation in the methylation status patterns of repetitive sequences. Sylwan.

[66] Deininger P.L., Moran J.V., Batzer M.A., Kazazian H.H. (2003). Mobile elements and mammalian genome evolution. Curr. Opin. Genet. Dev..

[67] Ehrlich M. (2002). DNA hypomethylation, cancer, the immunodeficiency, centromeric region instability, facial anomalies syndrome and chromosomal rearrangements. J. Nutr..

[68] Lee C., Wevrick R., Fisher R., Ferguson-Smith M., Lin C. (1997). Human centromeric dnas. Hum. Genet..

[69] Weiner A.M. (2002). Sines and lines: The art of biting the hand that feeds you. Curr. Opin. Cell Biol..

[70] Weisenberger D.J., Campan M., Long T.I., Kim M., Woods C., Fiala E., Ehrlich M., Laird P.W. (2005). Analysis of repetitive element DNA methylation by methylight. Nucleic Acids Res..

[71] Nikolaidis G., Raji O.Y., Markopoulou S., Gosney J.R., Bryan J., Warburton C., Walshaw M., Sheard J., Field J.K., Liloglou T. (2012). DNA methylation biomarkers offer improved diagnostic efficiency in lung cancer. Cancer Res..

[72] Liloglou T., Bediaga N.G., Brown B.R., Field J.K., Davies M.P. (2014). Epigenetic biomarkers in lung cancer. Cancer Lett..

[73] Anter J., Romero-Jimenez M., Fernandez-Bedmar Z., Villatoro-Pulido M., Analla M., Alonso-Moraga A., Munoz-Serrano A. (2011). Antigenotoxicity, cytotoxicity, and apoptosis induction by apigenin, bisabolol, and protocatechuic acid. J. Med. Food.

[74] Roman-Gomez J., Jimenez-Velasco A., Agirre X., Castillejo J.A., Navarro G., San Jose-Eneriz E., Garate L., Cordeu L., Cervantes F., Prosper F. (2008). Repetitive DNA hypomethylation in the advanced phase of chronic myeloid leukemia. Leuk. Res..

[75] Fukuwatari T., Kuzuya M., Satoh S., Shibata K. (2009). Effects of excess vitamin b1 or vitamin b2 on growth and urinary excretion of water-soluble vitamins in weaning rats. Shokuhin Eiseigaku Zasshi J. Food Hyg. Soc. Jpn..

[76] Oettel H., Frohberg H., Nothdurft H., Wilhelm G. (1965). Die prüfung einiger synthetischer farbstoffe auf ihre eignung zur lebensmittelfärbung. Arch. Toxicol..

[77] Borzelleca J., Hogan G. (1985). Chronic toxicity/carcinogenicity study of fd & c blue no. 2 in mice. Food Chem. Toxicol..

[78] Borzelleca J., Hallagan J. (1987). Lifetime toxicity/carcinogenicity study of fd & c red no. 3 (erythrosine) in mice. Food Chem. Toxicol..

[79] Tsujita J. (1979). Comparison of protective activity of dietafy fiber against the toxicities of various food colors in rats. Nutr. Rep. Int..

[80] Hansen W., Fitzhugh O., Nelson A., Davis K. (1966). Chronic toxicity of two food colors, brilliant blue fcf and indigotine. Toxicol. Appl. Pharmacol..

[81] Scotter M., Castle L. (2004). Chemical interactions between additives in foodstuffs: A review. Food Addit. Contam..

[82] Maekawa A., Matsuoka C., Onodera H., Tanigawa H., Furuta K., Kanno J., Jang J., Hayashi Y., Ogiu T. (1987). Lack of carcinogenicity of tartrazine (fd & c yellow no. 5) in the f344 rat. Food Chem. Toxicol..

[83] Moutinho I., Bertges L., Assis R. (2007). Prolonged use of the food dye tartrazine (fd&c yellow n° 5) and its effects on the gastric mucosa of wistar rats. Braz. J. Biol..

[84] Ford G., Gopal T., Grant D., Gaunt I., Evans J., Butler W. (1987). Chronic toxicity/carcinogenicity study of carmine of cochineal in the rat. Food Chem. Toxicol..

[85] Hiasa Y., Ohshima M., Kitahori Y., Konishi N., Shimoyama T., Sakaguchi Y., Hashimoto H., Minami S., Kato Y. (1988). The promoting effects of food dyes, erythrosine (red 3) and rose bengal b (red 105), on thyroid tumors in partially thyroidectomized n-bis (2-hydroxypropyl)-nitrosamine-treated rats. Jpn. J. Cancer Res..

[86] Collins T., Long E. (1976). Effects of chronic oral administration of erythrosine in the mongolian gerbil. Food Cosmet. Toxicol..

[87] Masannat Y.A., Hanby A., Horgan K., Hardie L.J. (2009). DNA damaging effects of the dyes used in sentinel node biopsy: Possible implications for clinical practice. J. Surg. Res..

[88] Davies J., Burke D., Olliver J., Hardie L., Wild C., Routledge M. (2006). The Induction of DNA Damage by Methylene Blue but Not by Indigo Carmine in Human Colonocytes In Vitro and In Vivo, Mutagenesis.

[89] Tsuda S., Murakami M., Matsusaka N., Kano K., Taniguchi K., Sasaki Y.F. (2001). DNA damage induced by red food dyes orally administered to pregnant and male mice. Toxicol. Sci..

[90] Durnev A., Oreshchenko A., Kulakova A. (1995). Analysis of cytogenetic activity of food dyes. Vopr. Meditsinskoi khimii.

[91] Miyachi T., Tsutsui T. (2005). Ability of 13 chemical agents used in dental practice to induce sister-chromatid exchanges in syrian hamster embryo cells. Odontology.

[92] Bhargav D., Singh M.P., Murthy R.C., Mathur N., Misra D., Saxena D.K., Chowdhuri D.K. (2008). Toxic potential of municipal solid waste leachates in transgenic *Drosophila melanogaster* (hsp70-lacz): Hsp70 as a marker of cellular damage. Ecotoxicol. Environ. Saf..

[93] Coulom H., Birman S. (2004). Chronic exposure to rotenone models sporadic parkinson’s disease in *Drosophila melanogaster*. J. Neurosci..

[94] Dean B.J. (1985). Recent findings on the genetic toxicology of benzene, toluene, xylenes and phenols. Mutat. Res. Genet. Toxicol..

[95] Hosamani R. (2013). Acute exposure of *Drosophila melanogaster* to paraquat causes oxidative stress and mitochondrial dysfunction. Arch. Insect Biochem. Physiol..

[96] Siddique Y.H., Fatima A., Jyoti S., Naz F., Khan W., Singh B.R., Naqvi A.H. (2013). Evaluation of the toxic potential of graphene copper nanocomposite (gcnc) in the third instar larvae of transgenic *rDosophila melanogaster* (hsp70-lacz) bg 9. PLoS ONE.

[97] Lloyd T.E., Taylor J.P. (2010). Flightless flies: *Drosophila* models of neuromuscular disease. Ann. N. Y. Acad. Sci..

[98] Collins S.J. (1987). The hl-60 promyelocytic leukemia cell line: Proliferation, differentiation, and cellular oncogene expression. Blood.

